# Analysis of volatile organic compounds released from human lung cancer cells and from the urine of tumor-bearing mice

**DOI:** 10.1186/1475-2867-12-7

**Published:** 2012-02-24

**Authors:** Yosuke Hanai, Ken Shimono, Hiroaki Oka, Yoshinobu Baba, Kunio Yamazaki, Gary K Beauchamp

**Affiliations:** 1 FRIST Research Center for Innovative Nanobiodevice, Nagoya University, Nagoya 464-8603, Japan; 2 Bioscience Technology Development Office, Panasonic Corporation, Kyoto 619-0237, Japan; 3 Department of Applied Chemistry, Graduate School of Engineering, Nagoya University, Nagoya 464-8603, Japan; 4 Monell Chemical Senses Center, Philadelphia, PA 19104-3308, USA

**Keywords:** Lung cancer, VOCs, GC-TOF MS, Cell medium, Urine, Tumor-bearing mice

## Abstract

**Backgrounds:**

A potential strategy for the diagnosis of lung cancer is to exploit the distinct metabolic signature of this disease by way of biomarkers found in different sample types. In this study, we investigated whether specific volatile organic compounds (VOCs) could be detected in the culture medium of the lung cancer cell line A549 in addition to the urine of mice implanted with A549 cells.

**Results:**

Several VOCs were found at significantly increased or decreased concentrations in the headspace of the A549 cell culture medium as compared with the culture medium of two normal lung cell lines. We also analyzed the urine of mice implanted with A549 cells and several VOCs were also found to be significantly increased or decreased relative to urine obtained from control mice. It was also revealed that seven VOCs were found at increased concentrations in both sample types. These compounds were found to be dimethyl succinate, 2-pentanone, phenol, 2-methylpyrazine, 2-hexanone, 2-butanone and acetophenone.

**Conclusions:**

Both sample types produce distinct biomarker profiles, and VOCs have potential to distinguish between true- and false-positive screens for lung cancer.

## Background

A 2008 report issued by the WHO [1], estimates that about 18% of all of cancer deaths can be attributed to lung cancer. Annually, lung cancer accounts for about 1.3 million deaths worldwide. For this reason, the development of a novel diagnostic test, which can facilitate the early detection of lung cancer, has the ability to vastly reduce lung cancer mortality rates. Although many studies report the utility of diagnostic imaging such as X-ray and CT scan, these modalities are both expensive and susceptible to false positive and false negative results.

The ability to combine imaging techniques with other methodologies such as biomarkers is a strategy with the potential to enhance the detection of lung cancer [2-4]. Although various biomarkers from blood, saliva and urine have been detected, including proteins, tumor antigens, anti-tumor antibodies, cell type-specific peptides, metabolic products and epigenetic phenomena such as hyper-methylated DNA, RNA, and the expression of specific genes [5], to date none of these biomarkers has had the adequate sensitivity, specificity and reproducibility to be considered for use clinically.

The analysis of exhaled breath for endogenous volatile organic compounds (VOCs) is one possible example of a non-invasive diagnostic assay that can be applied to cancer patients [6-9]. Current examples include the ^13^C-urea breath test for the detection of *Helicobacter pylori *[10,11], and the hydrogen-based breath test for carbohydrate malabsorption [12]. Another example is acetone, which is found at increased concentrations in the exhaled breath of patients with uncontrolled diabetes mellitus [13].

Sulfur-containing compounds such as ethylmercaptane, dimethylsulfide and dimethyldisulfide contribute to the characteristic odor of patients with liver cirrhosis [14]. In contrast, nitrogen-containing compounds are typical of patients suffering from uremia [15]. Ethane and pentane increase in concentration alongside the concentration of lipid peroxidation [16,17].

Several studies have shown that potential biomarkers for lung cancer are low molecular weight VOCs, which can be detected in the breath of lung cancer patients [18-24]. For example, a recent study using solid phase micro-extraction followed by gas chromatography showed that 1-butanol and 3-hydroxy-2-butanone is found at significantly higher concentrations in the breath of lung cancer patients compared with the control group [25]. Dragonieri et al. reported the use of an "electronic nose" which was able to discriminate between patients with lung cancer versus those with chronic obstructive lung disease (COPD) with a relatively high degree of sensitivity and specificity [26].

In addition to exhaled breath, urine is also considered to be a potential source of VOCs. However, one issue associated with the analysis of urine is the potential that VOCs detected in urine samples are derived from exogenous sources such as diet or the environment rather than as a result of the disease. For instance, Willis et al. reported that dogs could be trained to distinguish patients with bladder cancer on the basis of urine odor [27]. However, a follow-up study was unable to reproduce these findings in urine samples from patients with breast and prostate cancer [28]. Matsumura et al. also reported that sensor mice could be trained to discriminate between mice with and without tumors demonstrating that volatile odorants can be used to identify tumor-bearing mice [29]. Thus, there is mounting evidence that clinically relevant biomarkers for cancer may be found in urine.

Nevertheless, the cellular and biochemical origin of endogenous VOCs that have potential as lung cancer biomarkers are not well understood. A number of articles have investigated the release of VOCs from human cancer cells *in vitro *[25,30-32]. In the human lung carcinoma cell CALU-1, the release of branched hydrocarbons such as 2,3,3-trimethylpentane, 2,3,5-trimethylhexane, 2,4-dimethylheptane and 4-methyloctane were all found to be increased, whereas levels of acetaldehyde, 3-methylbutanal, *n*-butyl acetate, acetonitrile, acrolein, methacrolein, 2-methylpropanal, 2-butanone, methyl *tert*-butyl ether and hexanal were all decreased [31]. In the human lung cancer cell line NCI-H2087, alcohol 2-ethyl-1-hexanol and alkane 2-methylpentane were found at increased concentrations, compared with acetaldehyde, 2-methylpropanal, 3-methylbutanal, 2-methylbutanal and *n*-butyl acetate concentration which were found to be decreased [32]. While it has been reported that there may be a correlation between the VOCs detected in cell culture medium and in exhaled breath [25], no VOCs common to both urine and cell culture medium have been detected.

In the present study, we compared the VOCs released from the human lung adenocarcinoma cell line A549 to two normal lung cell lines, OUS-11 and WI-38 VA1 to determine whether any cancer specific compounds could be detected. We also investigated whether urine specific VOCs could be used to distinguish between tumor-bearing mice and healthy control mice, and whether there were any common compounds detected in both sample types.

## Result

### Comparative analysis of VOCs in the human lung cancer and normal lung cell lines

**Figure 1:**
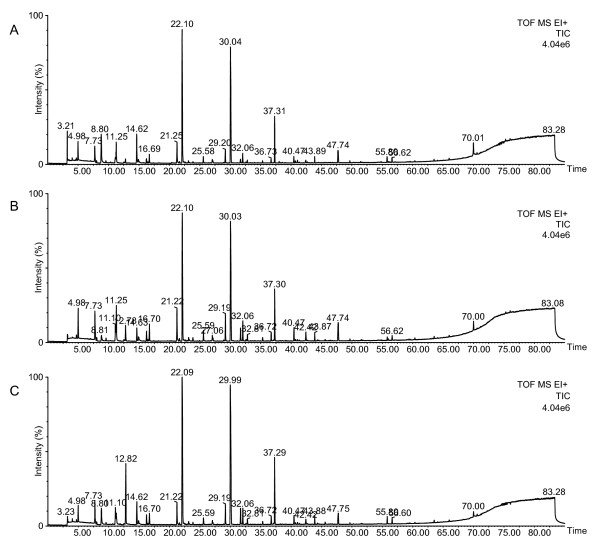
**Typical TIC of the VOCs from the culture medium samples of WI-38 VA13 cells (A), OUS-11 cells (B) and A549 cells (C) after 1-week incubation**. The TICs were obtained from analysis of the samples (200 μL) by HS-SPME (DVB/CAR/PDMS, 50/30 μm, 2 cm) and GC-TOF MS. The extraction temperature was 45°C and the time was 50 min. Desorption was performed at 240°C for 10 min. The injection was pulsed splitless (closed 3 min) with a 0.75 mm liner. Temperature programming consisted of an initial temperature 40°C for 5 min, followed by 3°C/min to 240°C with a 5 min hold at this final temperature. The other GC-MS conditions are described in the Material and Methods.

To extract the VOCs, upon reaching confluency the cell lines were grown for one, two and three weeks. After a one week incubation period, no dead cells were observed in any of the cell lines. After two week, some floating cells were observed and the culture medium had turned red (all cell lines). After the three week incubation period, many floating cells were observed and in all cell lines the culture medium was becoming amber. Thus, the culture conditions of the one and two week incubation periods ensured that the release of VOCs into the medium was mostly due to living cells. However, following three week incubation, cell viability diminished, indicating that released VOCs were not derived from the living cells. Typical total ion chromatograms (TICs) of VOCs measured in the medium samples of each cell line after one week incubation are shown in Figure 1. A sample of media obtained from the A549 cell lines showed a peak at 12.82 min that was clearly increased when compared with the control cell lines. However, the measured TICs were difficult to analyze because of multiple ion peaks, making it difficult to find the different EIC peaks in each of the cell lines. To analyze in more detail, comparative analysis between the A549 cell line and control cell lines was performed using XCMS software [33] and statistical analyses. Under our experimental conditions, an average of 751 ion peaks per sample was detected in the culture medium by XCMS software and statistical analyses. The significantly increased or decreased ion peaks (with a *p*-value < 0.10 and 1.5-fold higher or lower than average peak area of the normal control) were selected for identification. These ion peaks were summarized by deconvolution analysis of retention time and identified by spectral library match using the NIST'08 and Wiley library. However, identification of the VOCs was done only by means of spectral library matching (higher than 80% match) without confirmation of their retention times and comparison against a commercially available standard reagent. In this study, any peaks that could not be determined due to too low a library match were disregarded. The summary of determined VOCs by XCMS analysis are listed in Table 1, which also shows the rate of change with averaged peak area (using major *m/z*), comparing between the A549 and control cells. After a one week incubation period, there was a difference in the concentration of 31 compounds in the A549 cells compared with the control cells, whereby 18 compounds were found to be significant increased and 13 compounds were found to be significantly decreased (*p *< 0.10) (Table 1A). After a two week incubation period differences in the concentration of 45 compounds between the A549 and control cells culture medium were detected whereby 31 compounds were significantly increased, and 14 compounds were significantly decreased (*p *< 0.10) (Table 1B). After the three week incubation different concentrations were observed in 30 compounds, whereby 28 compounds were significant increased, and two compounds were significant decreased (*p *< 0.10) (Table 1C).

**Table 1:** List of the VOCs obtained from the culture medium of A549 cell line that were increased or decreased

A				
**Increased compound**	**CAS No**.	**Classification**	**fold**	***p*-value**

1-Dodecanol	112-53-8	alcohols	++	< 0.01
1-Methoxy-2-propanol	107-98-2	ethers	+++	0.02
2,2,4-Trimethyl-1,3-pentanediol diisobutyrate	6846-50-0	esters	+	0.09
2,5-Hexanedione	110-13-4	ketones	+	0.08
2-Butanone	78-93-3	ketones	+	0.06
2-Phenyl-2-propanol	617-94-7	alcohols	+++	0.02
3,3,5-Trimethylcyclohexanone	873-94-9	ketones	++	0.04
3-Butene-2-one	78-94-4	ketones	+	0.03
4-Cyanocyclohexene	100-45-8	nitriles	+	0.07
Acetonitrile	75-05-8	nitriles	+	0.03
Cyclohexanone	108-94-1	ketones	+	0.04
Diethyl ether	60-29-7	ethers	++	< 0.01
Dimethyl succinate	106-65-0	esters	++	0.06
Ethanol	64-17-5	alcohols	+++	0.01
Isobutyric acid 2-ethyl-3-hydroxyhexyl ester	74367-31-0	esters	+	< 0.01
Isophorone	78-59-1	ketones	++	0.02
Orthoformic acid tri-*sec*-butyl ester	16754-48-6	esters	+++	0.02
*tert*-Butanol	75-65-0	alcohols	+	< 0.01

Decreased compound	CAS No.	Classification	fold	*p*-value

1,3-Di-*tert*-butylbenzene	1014-60-4	hydrocarbons	---	0.07
2,4-Dimethyl-1-heptene	19549-87-2	hydrocarbons	---	0.04
2,4-Di-*tert*-butylphenol	96-76-4	phenols	-	0.06
2,5-Dimethyl-2,5-hexanediol	110-03-2	alcohols	--	0.02
2-Methyl-1-propanol	78-83-1	alcohols	--	0.06
4,6-Dimethyl-2-heptanone	19549-80-5	ketones	--	0.05
5-Methylnonane	15869-85-9	hydrocarbons	-	0.01
Acetophenone	98-86-2	ketones	-	0.08
Benzophenone	119-61-9	ketones	---	0.04
Benzyl alcohol	100-51-6	alcohols	--	0.02
Dichloromethane	75-09-2	halogens	-	< 0.01
Maltol	118-71-8	pyrans	---	< 0.01
Styrene	100-42-5	hydrocarbons	--	0.05

**B**				

Increased compound	CAS No.	Classification	fold	*p*-value
1-Dodecanol	112-53-8	alcohols	+++	< 0.01
2,2,4-Trimethyl-1,3-pentanediol diisobutyrate	6846-50-0	esters	+	0.03
2,2,5,5-Tetramethyltetrahydrofuran	15045-43-9	furans	+	< 0.01
2,2-Dimethyloxetane	6245-99-4	ethers	+	< 0.01
2,3-Dihydro-4-methylfuran	34314-83-5	furans	+	< 0.01
2,4,6-Trimethylpyridine	108-75-8	pyridines	+	0.01
2,4-Di-*tert*-butylphenol	96-76-4	phenols	+	0.01
2,5-Dimethyl-2,5-hexanediol	110-03-2	diols	+	0.03
2-Butanone	78-93-3	ketones	++	< 0.01
2-Hexanone	591-78-6	ketones	+	0.04
2-Methoxyfuran	25414-22-6	furans	+	0.02
2-Methyl-1-propanol	78-83-1	alcohols	+	0.02
2-Octanone	111-13-7	ketones	+	0.03
2-Pentanone	107-87-9	ketones	+	< 0.01
3-Aminopyrazole-4-carboxylic acid	24447-68-5	carboxylic acids	+	< 0.01
3-Methyl-3-buten-1-ol	763-32-6	alcohols	+	0.01
4-Isopropoxy-2-butanone	32541-58-5	ketones	+	0.02
4-Methylbenzyl alcohol	589-18-4	alcohols	+	< 0.01
Acetonylacetone	110-13-4	ketones	++	< 0.01
Acetophenone	98-86-2	ketones	++	< 0.01
Diethyl ether	60-29-7	ethers	++	< 0.01
Dimethyl succinate	106-65-0	esters	+++	< 0.01
DL-1-Phenylethyl alcohol	98-85-1	alcohols	+	0.01
Ethanol	64-17-5	alcohols	++	0.01
Furfural	98-01-1	aldehydes	+	0.02
Isobutyric acid 2-ethyl-3-hydroxyhexyl ester	74367-31-0	esters	+	0.01
Methyl vinyl ketone	78-94-4	ketones	+	< 0.01
Orthoformic acid tri-*sec*-butyl ester	16754-48-6	esters	+	< 0.01
*o*-Xylene	95-47-6	hydrocarbons	+	0.05
Pyrrole	109-97-7	pyrroles	++	< 0.01
*trans*-4-Methyl-2-pentene	674-76-0	hydrocarbons	+	0.01

Decreased compound	CAS No.	Classification	fold	*p*-value

1,2-Dihydro-2,2,4-trimethylquinoline	147-47-7	quinolines	-	0.01
1-Butanol	71-36-3	alcohols	-	0.05
2,3-Butanediol	19132-06-0	diols	-	< 0.01
2-Ethyl-1-hexanol	104-76-7	alcohols	-	0.06
2-Methyl-2-hepten-6-one	110-93-0	ketones	--	< 0.01
3,5-Lutidine	591-22-0	pyridines	-	< 0.01
Acetonitrile	75-05-8	nitriles	--	< 0.01
Benzophenone	119-61-9	ketones	--	< 0.01
Cyclohexanone	108-94-1	ketones	-	0.01
Dimethyl disulfide	624-92-0	Sulfurs	---	0.09
Isophorone	78-59-1	ketones	-	0.02
Maltol	118-71-8	pyrans	---	< 0.01
Methanethiol	74-93-1	thiols	---	0.1
Tributyl phosphate	126-73-8	esters	--	0.03

**C**				

Increased compound	CAS No.	Classification	fold	*p*-value
1,4-Diacetylbenzene	1009-61-6	ketones	++	< 0.01
1-Hydroxycyclohexanecarboxylic acid	1123-28-0	carboxylic acids	+++	< 0.01
2,2,4-Trimethyl-1,3-pentanediol diisobutyrate	6846-50-0	esters	++	< 0.01
2,4,6-Trimethylpyridine	108-75-8	pyridines	++	0.02
2,6-Di-*tert*-butylphenol	128-39-2	phenols	+	0.08
2-Butanone	78-93-3	ketones	++	< 0.01
2-Octanone	111-13-7	ketones	+	< 0.01
3-Methyl-3-buten-1-ol	763-32-6	alcohols	+	0.01
3-Octanol	589-98-0	alcohols	+	0.01
4-Cyanocyclohexene	100-45-8	nitriles	+	< 0.01
4-Methylbenzyl alcohol	589-18-4	alcohols	++	< 0.01
Acetophenone	98-86-2	ketones	+	< 0.01
Benzaldehyde	100-52-7	aldehydes	+	0.07
Benzophenone	119-61-9	ketones	+++	< 0.01
Benzyl alcohol	100-51-6	alcohols	++	< 0.01
Bisisobutyric acid 2,4,4-trimethylpentane-1,3-diyl ester	74381-40-1	esters	++	0.02
Carbamic acid, 4-methylphenyl ester	1850-13-1	esters	+++	< 0.01
Cyclohexanone	108-94-1	ketones	+	0.02
Diethyl ether	60-29-7	ethers	++	< 0.01
Dimethyl succinate	106-65-0	esters	+++	0.01
DL-1-Phenylethyl alcohol	98-85-1	alcohols	++	< 0.01
Ethanol	64-17-5	alcohols	++	0.09
Furfural	98-01-1	aldehydes	+	< 0.01
Isobutyric acid 2-ethyl-3-hydroxyhexyl ester	74367-31-0	esters	++	< 0.01
Phenol	108-95-2	phenols	+	0.04
*p*-Tolualdehyde	104-87-0	aldehydes	+	< 0.01
Pyrrole	109-97-7	pyrroles	+	< 0.01
*omega*-Caprolactam	105-60-2	amides	++	< 0.01

Decreased compound	CAS No.	Classification	fold	*p*-value

2,4-Dimethyl-1-heptene	19549-87-2	hydrocarbons	--	0.01
Maltol	118-71-8	pyrans	---	0.01

A: One week incubation; B: Two week incubation; C: Three week incubation

This table shows the VOCs, which were significantly different (*p *< 0.10) and more than 1.5 fold different to the averaged peak area (n = 3). The fold change indicates the difference in the averaged peak area between the A549 human lung cancer cell line and normal lung cells (+++ > 3 fold increase, ++ > 2 fold increase, + > 1.5 fold increase, --- < 3 fold decrease,

-- < 2 fold decrease, - < 1.5 fold decrease)

### Preparation of the human lung cancer mouse model

**Figure 2:**
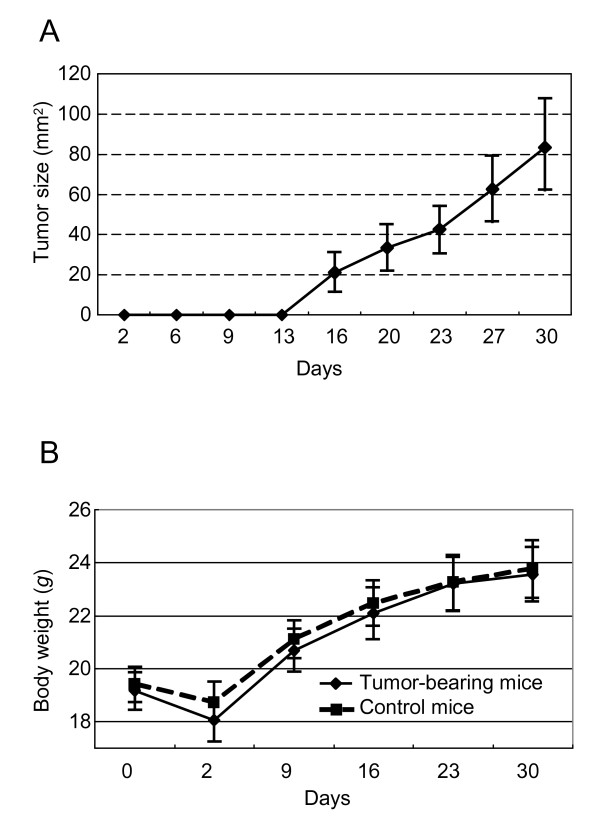
**Tumor measurement and animal body weight in the lung cancer animal model**. (**A**) Average tumor size in the tumor-bearing mice. Tumor size was calculated by multiplying the shortest and longest diameters using digital calipers. (**B**) The body weight of the tumor-bearing (solid line) and control mice (dash line). Each data point indicates an average value with standard deviation. The days indicate the number of days after transplantation. Body weight and tumor size of each mouse was recorded every few days.

To observe the proliferation of the A549 cells implanted into the mice, tumor size was measured (Figure 2a), with measurement commencing at day 13 post implantation. The body weight of the mice was also recorded (Figure 2b). Although there was a slight decrease in body weight in the two days following transplantation, this then gradually increased over subsequent days. There was no significant difference in body weight between the tumor-bearing and control mice.

### Comparative analysis of VOCs from the urine of tumor-bearing and control mice

**Figure 3:**
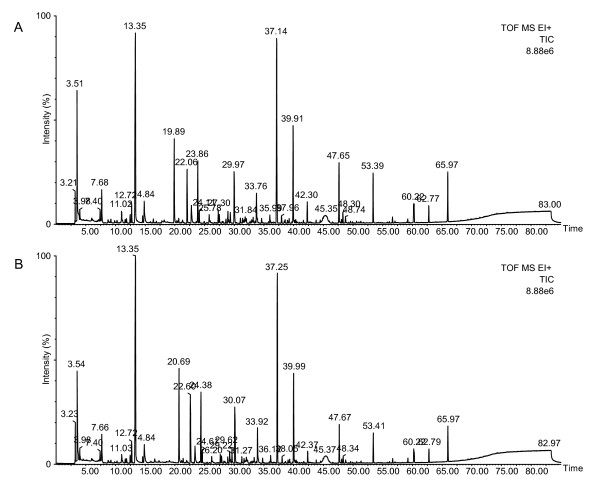
**Typical TIC of the VOCs from the urine samples obtained from the tumor-bearing (A) and normal control mice (B)**. The TIC was obtained from the analysis of urine samples (200 μL) by HS-SPME (DVB/CAR/PDMS, 50/30 μm, 2 cm) and GC-TOF MS. The extraction temperature was 45°C and the time was 50 min. Desorption was performed at 240°C for 10 min. The injection was pulsed splitless (closed 3 min) with a 0.75 mm liner. Temperature programming was set at an initial temperature of 40°C for 5 min, then programmed at 3°C/min to 240°C with a 5 min hold at this final temperature. All other GC-MS conditions are described in the Material and Methods.

The pooled urine samples from the tumor-bearing (n = 24) and control (n = 16) mice were analyzed by headspace solid-phase microextraction (HS-SPME) and gas chromatography time-of-flight mass spectrometry (GC-TOF MS). Two typical TICs of the VOCs obtained from the urine samples are shown in Figure 3. The chromatographs obtained from urine samples were very similar to the cultured medium samples, making it difficult to identify differences between peaks in EICs of the tumor-bearing and control mice. However, the results obtained from detailed and comparative analyses of the two groups did reveal some differences. On average the chromatogram from each urine sample contained 1493 ion peaks and from this 68 ion peaks were significantly increased and 65 ion peaks were decreased relative to the control group (*p *< 0.10 and characterized by a 1.5-fold higher or lower than average peak area of the control). Deconvolution analysis of the retention time of each peak allowed for the isolation of 76 VOCs that were significantly increased and six VOCs that were decreased. The isolated VOCs were picked up by similarity searching using the mass spectral library (NIST'08 and Wiley). Any VOC that could not be determined because a library match is too low was disregarded. Table 2 shows the 43 VOCs that were found be highly similar following a search of the database. However, these VOCs were identified only by means of spectral library match (higher than 80% match) without confirmation of their retention times and mass spectral by comparison against a commercially available standard reagent. Moreover, Table 2 shows the rate of change with averaged peak area (using major *m/z*), comparing between the tumor-bearing and control mice. In the urine obtained from the tumor-bearing mice, the concentration of 43 compounds was different relative to the control group (*p *< 0.10), whereby 42 compounds were significantly increased, and one compound was significantly decreased.

**Table 2:** List of the VOCs obtained from the tumor-bearing mice that were increased or decreased relative to the control group.

Increased compound	**CAS No**.	Classification	fold	*p*-value
1,3-Di-*tert*-butylbenzene	1014-60-4	ethers	+	0.08
2-(*sec*-butyl)-4,5-dihydrothiazole	56367-27-2	sulfurs	++	0.01
2,4-Dimethylheptane	2213-23-2	hydrocarbons	+	< 0.01
2,5-Dimethylpyrazine	123-32-0	pyrazines	+	0.07
2,5-Heptanedione	1703-51-1	ketones	+	0.03
2-Acetyl-5-methylfuran	1193-79-9	furans	+	0.02
2-Aminobenzamide	88-68-6	aldehydes	+	0.05
2-Butanone	78-93-3	ketones	+	0.02
2-Ethyl-5-methylfuran	1703-52-2	furans	+	0.06
2-Heptanone	110-43-0	ketones	+	0.04
2-Hexanone	591-78-6	ketones	+	0.03
2-Methoxy-5-methylthiophene	31053-55-1	sulfurs	+	0.02
2-Methylfuran	534-22-5	furans	++	0.01
2-Methylpyrazine	109-08-0	pyrazines	++	0.07
2-Pentanone	107-87-9	ketones	+	0.09
2-Methyl-6-vinylpyrazine	13925-09-2	pyrazines	++	0.02
3-Hexanone	589-38-8	ketones	+	0.1
3-Methyl-1-hexen-3-ol	55145-28-3	alcohols	++	0.01
4-Heptanone	123-19-3	ketones	+	0.03
4-Ketoisophorone	1125-21-9	ketones	+	0.05
4-Methoxyphenol	150-76-5	phenols	+	0.02
4-Methyloctane	2216-34-4	hydrocarbons	+	0.02
5-Hepten-2-one	6714-00-7	ketones	+	0.04
6-methyl-3-heptanone	624-42-0	ketones	+	0.03
Acetone	67-64-1	ketones	+	0.03
Acetophenone	98-86-2	ketones	+	0.03
Benzaldehyde	100-52-7	aldehydes	+	0.03
Dimethyl succinate	106-65-0	esters	+	0.05
Dimethyl trisulfide	3658-80-8	sulfurs	+	0.04
Dimethylamine	124-40-3	amines	+++	0.1
*exo*-Brevicomin	20290-99-7	ethers	+	0.03
*gamma*-Crotonolactone	497-23-4	esters	+	0.05
*gamma*-Hexanolactone	695-06-7	esters	+	0.02
Maltol	118-71-8	pyrans	+	0.02
*N*,*N*-Dimethylacetamide	127-19-5	amines	+	0.08
*N*-Benzylidenemethylamine	622-29-7	amines	+++	0.06
Phenol	108-95-2	phenols	+	0.01
Phenylacetone	103-79-7	ketones	+	0.07
*p*-Toluidine	106-49-0	amines	+	0.03
Pyrazinamide	98-96-4	amides	+	0.01
Quinazoline	253-82-7	Quinazolines	+++	0.07
Trimethylamine	75-50-3	amines	+	0.02

Decreased compound	CAS No.	Classification	fold	*p*-value

4-Methyl-6-hepten-3-one	26118-97-8	ketones	--	< 0.01

This table shows the VOCs, which were significantly different (*p *< 0.10) and more than 1.5 fold different to the averaged peak area (n = 3). The fold change indicates the difference in the averaged peak area between the tumor-bearing mice and normal control mice (+++ > 3 fold increase, ++ > 2 fold increase, + > 1.5 fold increase, --- < 3 fold decrease, -- < 2 fold decrease, - < 1.5 fold decrease).

### Seven VOCs were increased in both the A549 cell medium and urine from tumor-bearing mice

We also investigated the compounds in more detail that were increased in both the one and two week culture medium samples (A549 cells), and urine of the tumor-bearing mice. Seven compounds were increased in all three samples. It was also discovered that dimethyl succinate, phenol, 2-methylpyrazine, 2-hexanone, 2-butanone, 2-pentanone and acetophenone could be identified not only by spectral library match using NIST'08 and Wiley library but also by matching with retention time and mass spectral of commercially available reagents (data not shown).

To determine the concentration of the seven VOCs, a standard curve was prepared with analysis by HS-SPME. The standard curves were induced using the peak area at most abundance *m/z *value. The calibration measurements were used to determine the limit of detection (LOD) and subsequent quantification (LOD) of seven VOCs. Results in Table 3 present the accuracy of the standard curve. The standard curve of each VOC indicated good linearity in the range of 0.02 μM to 10 μM (R^2 ^= 0.99). The concentration of each VOCs was then calculated using the respective standard curves. The quantitative findings, with accompanying statistical analysis, are summarized in Tables 4 and 5 (cell cultures) and Table 6 (mice urine). The significance of the results was calculated by *ANOVA*.

**Table 3:** Summary of common VOCs from cells and mice

Compound	CAS	classification	Retention time of standard (min)	Quantified ion (*m/z*)	Range of calibration (μM)	R^2^	LOD (μM)	LOQ (μM)
2-Butanone	78-93-3	ketones	10.5	72	0.1-10	0.992	0.058	0.176
2-Pentanone	107-87-9	ketones	14.7	86	0.02-10	0.999	0.006	0.017
2-Hexanone	591-78-6	ketones	19.8	100	0.1-10	1.000	0.014	0.042
2-Methylpyrazine	109-08-0	pyrazines	27.0	94	0.05-10	1.000	0.004	0.013
Dimethyl Succinate	106-65-0	esters	36.3	115	0.1-10	1.000	0.014	0.043
Acetophenone	98-86-2	ketones	38.1	105	0.02-10	1.000	0.033	0.101
Phenol	108-95-2	phenols	45.6	94	0.1-10	1.000	0.011	0.033

Calibration were performed, giving the calibration curve, the limit of detection (LOD) and limit of quantitation (LOQ)

**Table 4:** Summary of VOCs detected in headspace of culture medium after one week incubation

Compound	OUS-11-1w		WI-38 VA13-1w		DMEM		A549-1w		A549 vs. OUS-11		A549 vs. WI-38 VA13		A549 vs. DMEM	
	
	Average (μM)	SD	Average (μM)	SD	Average (μM)	SD	Average (μM)	SD	*p*-value (ANOVA)	Ratio (A549/OUS-11)	*p*-value (ANOVA)	Ratio (A549/WI-38 VA13)	*p*-value (ANOVA)	Ratio (A549/DMEM)
2-Butanone	4.656	0.165	13.826	0.945	0.554	0.104	13.418	1.452	0.001	2.9	0.756	1.0	< 0.001	24.2
2-Pentanone	0.220	0.030	0.384	0.006	0.048	0.003	0.312	0.010	0.015	1.4	0.001	0.8	< 0.001	6.4
2-Hexanone	0.034	0.001	0.043	0.002	0.035	0.002	0.033	0.001	0.256	1.0	0.002	0.8	0.284	0.9
2-Methylpyrazine	< LOD		0.004	0.000	0.016	0.003	0.004	0.001	0.012	-	0.337	0.9	0.004	0.2
Dimethyl Succinate	0.046	0.001	0.045	0.001	< LOD		0.055	0.004	0.023	1.2	0.016	1.2	< 0.001	-
Acetophenone	0.403	0.026	0.647	0.019	< LOD		0.465	0.020	0.055	1.2	0.001	0.7	< 0.001	-
Phenol	0.099	0.002	0.160	0.003	0.041	0.001	0.095	0.020	0.835	1.0	0.011	0.6	0.019	2.3

**Table 5:** Summary of VOCs detected in headspace of culture medium after two week incubation

Compound	OUS-11-2w		WI-38 VA13-2w		DMEM		A549-2w		A549 vs. OUS-11		A549 vs. WI-38 VA13		A549 vs. DMEM	
	
	Average (μM)	SD	Average (μM)	SD	Average (μM)	SD	Average (μM)	SD	*p*-value (ANOVA)	Ratio (A549/OUS-11)	*p*-value (ANOVA)	Ratio (A549/WI-38 VA13)	*p*-value (ANOVA)	Ratio (A549/DMEM)
2-Butanone	4.293	0.184	8.971	0.761	0.767	0.120	14.616	0.570	< 0.001	3.4	0.001	1.6	< 0.001	19.1
2-Pentanone	0.158	0.003	0.203	0.024	0.031	0.002	0.363	0.016	< 0.001	2.3	0.001	1.8	< 0.001	11.6
2-Hexanone	0.030	0.000	0.033	0.000	0.034	0.002	0.037	0.002	0.008	1.2	0.039	1.1	0.150	1.1
2-Methylpyrazine	< LOD		0.004	0.001	0.015	0.003	0.004	0.000	< 0.001	-	0.750	1.0	0.005	0.3
Dimethyl Succinate	0.043	0.000	< LOD		< LOD		0.052	0.001	< 0.001	1.2	< 0.001	-	< 0.001	-
Acetophenone	0.245	0.004	0.311	0.015	< LOD		0.561	0.015	< 0.001	2.3	< 0.001	1.8	< 0.001	-
Phenol	0.077	0.006	0.120	0.005	0.031	0.001	0.129	0.005	0.001	1.7	0.144	1.1	< 0.001	4.1

The VOC concentrations are shown with standard deviations. The *p*-values were calculated by *ANOVA*. The ratio is a comparison of average concentrations of A549 and each samples

**Table 6:** Summary of VOCs detected in headspace of mice urine

Compound	CAS	Retention time (min)	control mice		tumor-bearing mice		*p*-value (ANOVA)	Ratio (tumor/control)
					
			Average (μM)	SD	Average (μM)	SD		
2-Butanone	78-93-3	10.5	8.808	1.154	13.226	1.297	0.02	1.5
2-Pentanone	107-87-9	14.7	2.180	0.209	2.599	0.142	0.08	1.2
2-Hexanone	591-78-6	19.8	0.085	0.016	0.134	0.010	0.02	1.6
2-Methylpyrazine	109-08-0	27.0	0.201	0.029	0.455	0.109	0.03	2.3
Dimethyl Succinate	106-65-0	36.3	0.237	0.017	0.334	0.038	0.03	1.4
Acetophenone	98-86-2	38.1	2.310	0.499	3.912	0.399	0.02	1.7
Phenol	108-95-2	45.6	1.824	0.207	2.722	0.213	0.01	1.5

The VOC concentrations are shown with standard deviations. The p-values were calculated by *ANOVA*. The ratio is a comparison of average concentrations of tumor-bearing mice and control mice

Summary of the concentration and significant difference of seven VOCs in culture medium were shown in Tables 4 and 5. After one week incubation, five VOCs in A549 cell medium were significantly increased (2-butanone, 2-pentanone, dimethyl succinate, acetophenone, phenol), 2-methylpyrazine was significantly decreased in comparison to the VOCs in the DMEM. Four VOCs in A549 cell medium were significantly increased in comparison to the VOCs in OUS-11 cell medium; 2-butanone (*p *= 0.001), 2-pentanone (*p *= 0.015), 2-methylpyrazine (*p *= 0.012), dimethyl succinate (*p *= 0.023). Five VOCs in A549 cell medium were significantly increased in comparison to the VOCs in WI-38 VA13 cell medium; 2-pentanone (*p *= 0.001), 2-hexanone (*p *= 0.002), dimethyl succinate (*p *= 0.016), acetophenone (*p *= 0.001), phenol (*p *= 0.011). After two week incubation, five VOCs in A549 cell medium were significantly increased (2-butanone, 2-pentanone, dimethyl succinate, acetophenone, phenol), 2-methylpyrazine was significantly decreased in comparison to VOCs in the DMEM. All seven VOCs in A549 cell medium were significantly increased in comparison to the VOCs in OUS-11 cell medium; 2-butanone (*p *< 0.001), 2-pentanone (*p *< 0.001), 2-hexanone (*p *= 0.008), 2-methylpyrazine (*p *< 0.001), dimethyl succinate (*p *< 0.001), acetphenone (*p *< 0.001) and phenol (*p *= 0.001). Five VOCs in A549 cell medium were significantly increased in comparison to the VOCs in WI-38 VA13 cell medium; 2-butanone (*p *= 0.001), 2-pentanone (*p *= 0.001), 2-hexanone (*p *= 0.039), dimethyl succinate (*p *< 0.001), acetphenone (*p *< 0.001).

Summary of the concentration and significant difference of all seven VOCs in mice urine was shown in Table 6. All VOCs were significantly increased in tumor-bearing mice urine in comparison to the VOCs in control mice urine; 2-butanone (*p *= 0.02), 2-pentanone (*p *= 0.08), 2-hexanone (*p *= 0.02), 2-methylpyrazine (*p *= 0.03), dimethyl succinate (*p *= 0.03), acetphenone (*p *= 0.02) and phenol (*p *= 0.01).

## Discussion

The ability to use the VOCs detected in the urine of patients with lung cancer as putative tumor markers may facilitate the rapid detection and non-invasive diagnosis of lung cancer. In this study, we identified VOCs derived from three cell lines (A549, OUS-11, WI-38 VA13) and from the urine of mice inoculated with the A549 human tumor cell line. The compounds were detected by HS-SPME and analyzed by GC-MS. The SPME method is simple and highly sensitive, and the in this study we used a SPME fiber (2 cm divinylbenzene/carboxen/polydimethylsiloxane: DVB/CAR/PDMS) which was the most absorbent as shown by the number of peaks which we were able to detect. Using this fiber, we analyzed the substances contained in the headspace of each sample.

Several studies have also reported that VOCs are associated with lung cancer [18,20-24]. For most of these VOCs however, the cancer cells from which they are derived and their biochemical origins were not determined, allowing for the possibility that they are exogenous substances. In this study, we searched for characteristic compounds derived from lung cancer cells by comparing the VOCs from lung cancer cells (A549) and non-tumor cells. Under our experimental conditions, the composition of the detected VOCs was different for each cell and further varied according to incubation period. Moreover, it was found that 18 (one week incubation), 31 (two week incubation) and 28 compounds (three week incubation) were increased in the culture medium of A549 cells compared with non-tumor cells. These compounds mostly consisted of ketones and alcohols. In the case of the one week incubation group, alcohols (1-methoxy-2-propanol, 2-phenyl-2-propanol and ethanol) were significantly increased, compared with the control cells. The two week incubation caused an increase in the concentration of 1-dodecanol and dimethyl succinate. In the case of the cells incubated for three week the concentration of 1-hydroxycyclohexanecarboxylic acid, benzophenone, carbamic acid 4-methylphenyl ester and dimethyl succinate were all increased. However, at the three week incubation period, many dead cells were observed and the color of medium was becoming amber, meaning that the VOCs obtained from this experimental group may not reflect VOCs derived from living cells. Ten of the VOCs in the one week and two week incubation period were found to be common to both groups: dimethyl succinate, diethyl ether, ethanol, 2,2,4-trimethyl-1,3-pentanediol diisobutyrate, isobutyric acid 2-ethyl-3-hydroxyhexyl ester, 2-butanone, 1-dodecanol, 3-butene-2-one, orthoformic acid tri-*sec*-butyl ester and 2,5-hexanedione. These include the plasticizer-like compounds (e.g. 2,2,4-Trimethyl-1,3-pentanediol diisobutyrate). Thus, it is necessary to consider the possibility that exogenous compounds were also detected. Filipiak et al. also reported that the concentration of ethanol was significantly increased in A549 cells, although this finding was made under different experiment conditions [34]. However, some of the VOCs identified in cultured medium of the A549 cells differ from the compounds reported by Filipiak. The culture conditions and extraction methods (thermal desorption vs solid-phase microextraction) may be responsible for this difference. It has also been reported that 2-butanone was increased in the cultured medium of NCI-H2087 and CALU-1 lung cancer cell lines, although not at remarkably high levels [31,32]. Additionally, Sponring et al. has also suggested that 2-butanone may be increased in the breath of lung cancer patients [32]. The reason for the differences in the release or consumption of VOCs among the investigated cell lines is currently unknown, but may result from phenotypic or genotypic differences. The differences may also reflect the characteristics of the specific cell lines tested.

We created a mouse model of human lung cancer, and analyzed the VOCs obtained from the urine of these mice. When the urinary VOCs of the tumor-bearing mice were compared with of the control group differences in 43 compounds was observed (42 were increased and one was decreased). Matsumura et al. created a mouse lung cancer model, and identified the characteristics of some VOCs from the urine of these animals, including that 2-heptanone, 5-heptene-2-one were able to distinguish between the lung cancer group and the control group [29]. According to our results, 2-heptanone and 5-heptene-2-one were identified as being significantly increased in the urine of the lung cancer group.

We also found that seven VOCs (dimethyl succinate, 2-pentanone, phenol, 2-methylpyrazine, 2-hexanone, 2-butanone, acetophenone) were increased in both the urine of the tumor-bearing mice and the culture medium of the A549 cells. These compounds were identified not only by spectral library matching with NIST'08 and the Wiley library but also by matching the retention times and mass spectrum of commercially available reagents. However, with respect to which culture period is the optimal condition with which to compare with the *in vivo *model, it is difficult to nominate a culture period that closely mimics the characteristics of the cancer cells in the mice 26-30 days after transplantation. The conditions of the two week culture period are expected to be similar to the tumor-bearing mice, based on the idea that it takes a few days from seeding the cells until they are confluent. 2-Butanone, 2-pentanone, dimethyl succinate, and acetophenone in A549 culture medium were significantly increased in comparison to other samples including DMEM only. So these five VOCs considered suitable candidates for cancer biomarkers. Since 2-methylpyrazine and 2-hexanone are not likely to be released from A549 cancer cell, we currently made a conclusion to discard these compounds from candidates for biomarker. O'Nell et al. reported that 2-butanone and acetophenone can be detected in the exhaled breath of lung cancer patients [35]. It has also been reported that benzaldehyde, 2-butanone and acetophenone are three of the compounds identified as potential breath markers for lung cancer, in a test which has 80% sensitivity and 100% specificity for the diagnosis of lung cancer [36].

Based on the results obtained from this study, it could be also suggested that some of the VOCs unique to lung cancer may also be detected in urine. Our results provide preliminary evidence that urinary biomarkers may also represent a feasible method for the early detection and non-invasive diagnosis of lung cancer.

## Methods

### Cell lines and culturing

Three different human cell lines were used in the experiments: A549 (JCRB No. JCRB0076), OUS-11 (JCRB No. JCRB1034), WI-38 VA13 (JCRB No. JCRB9057). The A549 cells were originally isolated from a lung carcinoma of a 58-y-old man and are characterized by the presence of a mutated K-ras but a wild-type B-raf gene. The OUS-11 and WI-38 VA13 cells were used as normal control cells for the purposes of comparison and were derived from normal tissue of lung cancer patient and from a SV40 virus transformed lung fibroblast cell line, respectively. The cell lines were obtained from the Health Science Research Resource Bank (Osaka, Japan). All cells have grown in DMEM high-glucose culture medium (Sigma-Aldrich Japan, Tokyo) supplemented with 10% fetal bovine serum (HyClone, Logan, UT), penicillin (30 unit/mL, Meiji Seika, Tokyo, Japan) and streptomycin (30 μg/mL, Meiji Seika, Tokyo, Japan). The cells were cultured in two culture dishes (100 mm × 20 mm) in 10 mL culture medium and every three days fresh medium was applied. For all experiments, the cells were cultured under standard conditions at 37°C in humidified atmosphere containing 5% CO_2_. For the VOC measurements, the A549, OUS-11 and WI-38 VA13 cells were incubated for one, two and three-weeks after all cell lines grew as monolayer adherent on the surface of culture dishes. After this incubation period, the culture medium was collected and stored at -80°C until analysis.

### Tumor model mice of A549 cell line and collection of urine

Five-week-old female C.B-17/lcr-scidJcl mice (CLEA Japan, Tokyo, Japan) were separated into two groups, those bearing tumors (n = 24) and those used as controls (n = 16). To establish the human tumor-bearing model, A549 cancer cells were prepared at a concentration of 1.0 × 10^8 ^cells/mL in PBS, and implanted subcutaneously into the right ventral flank area by injecting 0.1 mL of the suspension. The control mice were injected only with 0.1 mL PBS on the same position as tumor-bearing mice. The day of inoculation was defined as day 0. The mice were kept in glass-shielded metabolic cages (Metabolica; Sugiyamagen, Tokyo, Japan) under sterile conditions and were supplied with radiation sterilized feed CE-2 (CLEA Japan, Tokyo Japan) and autoclaved drinking water, which was provided *ad libitum*. After transplantation, urine was collected from the mice daily, and stored at -80°C until time of analysis. Body weight and tumor size (the shortest and longest diameters) of each mouse was recorded every few days. Tumor size was calculated by multiplying the length of the shortest and longest diameters. The mice were sacrificed 30 days after transplantation. The urine samples were collected from 26 to 30 days after transplantation.

All animals were treated in accordance with the institutional guidelines for the care and use of laboratory animals.

### Extraction of VOCs by headspace solid-phase microextraction

Prior to the analysis of the urine and culture medium, we sought to determine the optimal suitable SPME fiber. Four types of SPME fibers (carboxen/polydimethylsiloxane: CAR/PDMS, divinylbenzene/carboxen/polydimethylsiloxane: DVB/CAR/PDMS, polydimethylsiloxane/divinylbenzene: PDMS/DVB, Polyacrylate) were compared with the chromatogram obtained from the analysis of urine samples. The results of this analysis, revealed the 2 cm 50/30 μm DVB/CAR/PDMS (Supelco Corp, Bellefonte, PA, USA) fiber to be the optimal and this fiber was used for subsequent analyses. The urine and cell culture medium were centrifuged at 10,000 *g *for 5 min and, the supernatant was used for the analysis. The VOCs from urine and cell culture medium were extracted by HS-SPME methods using of Combi-pal auto sampler (CTC Analytics, Switzerland). A 200 mL sample of the urine or culture medium was applied to a 2 mL crimp top vial and sealed by crimp cap. The vial was set on Combi-pal and equilibrated for 10 min at 45°C. The volatile compounds in the headspace were extracted by SPME fiber for 50 min at 45°C.

### Gas chromatography and mass spectrometry

The GC-TOF MS system was composed of a 7890a GC (Agilent, CA, USA) equipped with an auto sampler Combi-pal and GCT Premier (Waters, MA, USA) for time of flight mass spectrometry. The SPME fiber with absorbed volatile compounds was inserted into the injection port of the 7890a GC using the auto sampler and desorbed for 10 min at 240°C. The injection was pulsed splitless (closed 3 min) with a 0.75 mm liner. The GC-TOF MS system was equipped with an InertCap Pure-WAX T.L. column (60 m + 2 m transfer line, 0.25 mm i.d., 0.5 μm thick; GL science, Japan), which was used for separation and analysis of the desorbed volatiles. We employed the following chromatographic protocol for separation before MS analyses: 40°C for 5 min, then programmed at 3°C/min to 240°C with a 5 min hold at this final temperature. Column flow was constant at rate of 1 ml/min. The injection port was held at 240°C. Operating parameters for the mass spectrometer were as follows: the ion source temperature was 200°C, ionizing energy at 70 eV, scanning frequency was 0.2 s/spectrum from *m/z *40 to *m/z *500. Peak identification was accomplished through manual interpretation of spectra and matching against the mass spectral library (NIST/EPA/NIH mass spectral library (NIST 08); mass spectral library of drugs, poisons, pesticides, pollutants and their metabolites Wiley, USA) and comparison with commercially available standard samples, which were purchased from the Tokyo Chemical Industry (Tokyo, Japan). Each medium sample was analyzed in triplicate.

### Data processing and quantitative analysis

The chromatographic peak areas were integrated using the MassLynx 4.1 (Waters, MA, USA). Genesis peak detection was applied for a list of putatively annotated ions. Detection and integration of generated ion peak from electron ionization were also performed using a XCMS software package version 1.16.3 (http://masspec.scripps.edu) [33], running under R version 2.10.1 (http://cran.r-project.org/). The significance levels of differences between groups were calculated using a student's *t*-test that come with XCMS software. We also determined that the "increased extracted ion peaks" had a *p*-value of < 0.10, and were 1.5 times higher than the averaged peak area of the other group. Of those peaks, a manual inspection of the EIC for each peak was made to validate the detected peak. Refine the VOC was performed by manual deconvolution based on retention time and peak shape of each increased extracted ion peak.

The identified VOCs were quantified using commercially available reagents. For each of the reagents, stock solutions were made to a concentration of 100 mM (Sigma-Aldrich, MI, USA) by dissolving them into 1 mL mixture of water and methanol (1/1 v/v). Calibration solutions of 0.01, 0.02, 0.05, 0.1, 0.5, 1.0, and 10.0 μM were also made up. The standard curves were created based on the peak areas, which were obtained from HS-SPME GC-TOF MS analysis of the calibration solution. The data were analyzed in triplicate. The *p*-values were obtained using ANOVA.

## Abbreviations

VOCs: volatile organic compounds; COPD: chronic obstructive lung disease; TIC: total ion chromatogram; HS-SPME: headspace solid-phase microextraction; GC-TOF MS: gas chromatography time-of-flight mass spectrometry; DVB/CAR/PDMS: divinylbenzene/carboxen/polydimethylsiloxane; CAR/PDMS: carboxen/polydimethylsiloxane; PDMS/DVB: polydimethylsiloxane/divinylbenzene

## Competing interests

The authors declare that they have no competing interests.

## Authors' contributions

YH carried out the experiments and participated in the design of the study. KS advised in the study design and helped to draft the manuscript. YB helped to draft the manuscript. HO, KY, and GKB conceived of the study, participated in its design and coordination, and helped to draft the manuscript. All authors read and approved the final manuscript.
